# The effectiveness of intensive mobilization technique combined with capsular distension for adhesive capsulitis of the shoulder

**DOI:** 10.1186/1757-1146-7-S1-A115

**Published:** 2014-04-08

**Authors:** Sunwook Park, Hansuk Lee

**Affiliations:** 1 Department of physical therapy, Eulji University, Seongnam, Gyeonggi, Korea

## Background

The management of Adhesive Capsulitis(AC) is controversal. Both capsular distension after intraarticular injection and mobilization therapy are known as highly effective treatment of painful and limited shoulder joint. The aim of this study was to determine the synergistic effect of intensive mobilization technique combined with capsular distension for patients with adhesive capsulitis in improving shoulder ROM, pain and function.

## Method

A total of 28 subjects suffering from AC were randomized into two groups. Group A received fluoroscopically guided capsular distension with steroid injection followed by intensive mobilization technique twice per week for 4 weeks and group B only received capsular distension with steroid injection.

## Results

Patients were assessed using the Shoulder Pain and Disability Index (SPADI), Constant-Murley Shoulder Function Assessment Score (CS), Active Range of Motion(AROM) and Verbal Numeric Score(VNS). All measurements were obtained before injection and mobilization and 4 weeks after the procedures in order to compare with effects of the treatments.

All measured value was significantly difference in group A and B(P<.05). Only external rotation ROM was not significantly difference in group B. The statistical differences were observed between group A and B in SPADI, CS, AROM and VNS (P<.01).

## Conclusions

Treatment using steroid injection with distension followed by intensive mobilization technique is recommended rather than injection treatment alone for the treatment of AC. In order to maximize the effectiveness of the two treatments further studies for optimal mobilization techniques will be needed.

